# Total Osteopontin and Its Isoform OPN4 Are Differently Expressed in Respiratory Samples during Influenza A(H1N1)pdm09 Infection and Progression

**DOI:** 10.3390/microorganisms11051349

**Published:** 2023-05-20

**Authors:** Jéssica Santa Cruz de Carvalho Martins, Thiago das Chagas Sousa, Maria de Lourdes de Aguiar Oliveira, Etel Rodrigues Pereira Gimba, Marilda Mendonça Siqueira, Aline da Rocha Matos

**Affiliations:** 1 Laboratório de Vírus Respiratórios, Exantemáticos, Enterovírus e Emergências Virais, Instituto Oswaldo Cruz, Fiocruz. Av. Leopoldo Bulhões, Manguinhos, 1480, Rio de Janeiro 20230-130, Brazil; 2 Grupo de Hemato-Oncologia Molecular, Coordenação de Pesquisa, Instituto Nacional de Câncer, Praça da Cruz Vermelha, 23, andar 6, Rio de Janeiro 20230-130, Brazil; 3 Programa de Pós-Graduação Stricto Sensu em Oncologia, Instituto Nacional de Câncer, Rua André Cavalcanti, 37, andar 3, Rio de Janeiro 20231-050, Brazil; 4 Programa de Pós-Graduação em Ciências Biomédicas, Fisiologia e Farmacologia, Instituto Biomédico, Av. Prof. Hernani Melo, 101, Niterói 24210-130, Brazil; 5 Departamento de Ciências da Natureza, Universidade Federal Fluminense, Rua Recife 1-7, Bela Vista, Rio das Ostras 28880-000, Brazil

**Keywords:** influenza, osteopontin, expression, biomarker

## Abstract

Influenza A virus (IAV) infection affects the human respiratory tract, causing an acute and highly contagious disease. Individuals with comorbidities and in the extremes of age are classified as risk groups for serious clinical outcomes. However, part of the severe infections and fatalities are observed among young healthy individuals. Noteworthy, influenza infections lack specific prognostic biomarkers that would predict the disease severity. Osteopontin (OPN) has been proposed as a biomarker in a few human malignancies and its differential modulation has been observed during viral infections. However, OPN expression levels in the primary site of IAV infection have not been previously investigated. Therefore, we evaluated the transcriptional expression patterns of total OPN (*tOPN*) and its splicing isoforms (*OPNa, OPNb, OPNc, OPN4*, and *OPN5*) in 176 respiratory secretion samples collected from human influenza A(H1N1)pdm09 cases and a group of 65 IAV-negative controls. IAV samples were differentially classified according to their disease severity. *tOPN* was more frequently detected in IAV samples (34.1%) when compared with the negative controls (18.5%) (*p* < 0.05), as well as in fatal (59.1%) versus non-fatal IAV samples (30.5%) (*p* < 0.01). *OPN4* splice variant transcript was more prevalent in IAV cases (78.4%) than in the negative controls (66.1%) (*p* = 0.05) and in severe cases (85.7%) in relation to the non-severe ones (69.2%) (*p* < 0.01). *OPN4* detection was also associated with severity symptoms such as dyspnea (*p* < 0.05), respiratory failure (*p* < 0.05), and oxygen saturation < 95% (*p* < 0.05). In addition, the *OPN4* expression level was increased in the fatal cases of respiratory samples. Our data indicated that *tOPN* and *OPN4* had a more pronounced expression pattern in IAV respiratory samples, pointing to the potential use of these molecules as biomarkers to evaluate disease outcomes.

## 1. Introduction

The influenza A virus (IAV) is a pathogen with a great importance for public health as it causes highly contagious acute respiratory infections that promote relevant annual seasonal epidemics worldwide. Influenza virus infections affect 3–5 million people in a serious way, and are responsible for about 300,000–650,000 deaths annually in the world [1,2]. Most IAV infections are clinically classified as mild, presenting with self-limited symptoms. However, a smaller fraction of the cases can develop severe symptoms with a further evolution to hospitalization and fatality. Individuals classified as a high risk for the development of IAV severe respiratory disease mainly include the elderly, children < 5 years old, pregnant women, and people with immune impairments and comorbidities [3]. These individuals are usually the target groups for vaccination campaigns and the administration of antiviral treatments, mainly performed with neuraminidase inhibitors such as oseltamivir [4,5,6,7]. 

This pathogen belongs to the *Orthomyxoviridae* family and its genetic material is composed of segmented RNA with a negative polarity [8]. It is divided into four types: A, B, C, and D. Of these, types A and B are frequent in annual epidemics, with type A being responsible for the most serious infections [9,10]. This is mainly due to the characteristic of this virus to infect hosts of multiple species, providing the reassortment of different RNA segments that culminates in the emergence of new strains with a pandemic potential, as happened in 2009 [11,12]. During the 2009 influenza A(H1N1) pandemic, a substantial number of healthy adults were severely affected by the infection [13], which may be partly explained by pathogen virulence factors [14]. Despite that, the genetic variation in human pivotal genes involved in the antiviral immune response and its implication in IAV poor disease outcomes has been investigated [15,16,17]. 

IAV infections trigger host antiviral mechanisms that modulate human signaling cascades that modify pro-inflammatory cytokines, including osteopontin (OPN). This protein is a member of the group of small integrin-binding ligand N-linked glycoproteins (SIBLING) and participates in distinct physiological and pathological conditions such as bone remodeling, inflammation, and oncogenesis [18,19]. OPN is produced by different cell types, including endothelial, epithelial, and immune cells, activating them in response to stimuli such as viral infections [19,20,21]. Previous reports demonstrated that OPN transcript levels were higher in the blood samples of IAV patients than in healthy controls [22]. Additionally, epithelial cells infected with respiratory syncytial virus (RSV) also presented an upregulated expression of OPN [21]. More recently, in cases of severe acute respiratory syndrome coronavirus 2 (SARS-CoV-2) infections, it was found that OPN serum levels were higher in patients with severe coronavirus disease 2019 (COVID-19) when compared with patients with a mild disease presentation [23]. Moreover, OPN expression was induced in human macrophages infected in vitro with dengue virus (DNV), and plasma levels were elevated in DNV-infected patients [24]. OPN expression levels were also increased in the brain tissues of animals infected with West Nile virus (WNV) [20,24].

**Figure 1:**
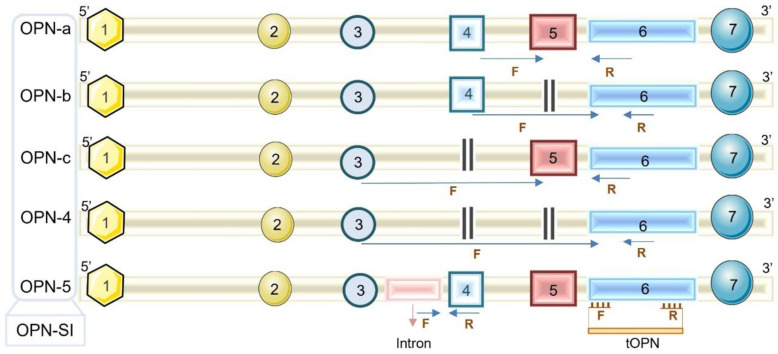
Representation of mRNA sequence of OPN isoforms. The numbers in the boxes represent the set of exons and introns that are present in the distinct OPN isoforms generated by alternative splicing (*OPNa*, *OPNb*, *OPNc*, *OPN4*, and *OPN5*). *tOPN* (total OPN) represents all the OPN transcripts as this includes the amplification of exon 6, common to all OPN transcripts. F: forward primer annealing site; R: reverse primer annealing site.

The OPN primary transcript undergoes alternative splicing, generating five splicing isoforms, named as *OPNa, OPNb, OPNc, OPN4,* and *OPN5*. The isoforms vary in the set of exons that form them or due to the presence of distinct introns [25] (Figure 1). In addition, OPN undergoes several post-translational modifications, including phosphorylation, proteolytic cleavage, sulfation, and glycosylation, which also leads to the formation of different isoforms, which together are total OPN (*tOPN*) or just OPN. Such modifications reflect the distinct functions of this protein [26,27] as well as its differential expression in various tissues and pathologies. The splicing isoforms are aberrantly expressed in malignancies such as ovarian, prostate, breast, and thyroid cancer [25,28,29], but their levels have not been investigated during respiratory viral infections. 

To address this knowledge gap, this work aimed to evaluate the expression patterns of *tOPN* and its isoforms in human respiratory specimens from IAV cases differentially classified according to their disease severity, including fatal cases. We also aimed to correlate *OPN* expression levels with the presence of respiratory symptoms associated with poor disease outcomes. Notably, we found that the presence of *tOPN* and *OPN4* transcripts was more frequent in respiratory samples from influenza A(H1N1)pdm09 cases when compared with healthy control samples. Additionally, fatal IAV cases had higher *OPN4* expression levels than influenza-like illness (ILI) and severe acute respiratory infection (SARI) groups. 

## 2. Materials and Methods

### 2.1. Respiratory Samples and Case Clinical Classification

Our laboratory is a National Influenza Center in Brazil and is, therefore, part of the World Health Organization (WHO) influenza surveillance network. Accordingly, it receives samples from 9 out of the 27 Brazilian States, from the Brazilian Northeastern, Southeastern, and Southern regions. Respiratory samples received during the 2016 and 2017 seasons were collected as nasopharyngeal swabs from 176 influenza A(H1N1)pdm09 patients, which were further classified according to their infection severity. ILI cases were defined as patients who presented mild symptoms such as fever, headaches, and coughs. SARI cases were classified as hospitalized patients who had dyspnea or respiratory failure, or oxygen saturation <95%. Among the total cases, we also included fatalities. In addition, respiratory samples from 65 individuals that presented with ILI symptoms but who were diagnosed as influenza-negative were used as a control group, which was included to demonstrate the specificity of the findings for influenza infections. However, their etiological agent was not investigated. Overall, 32.4% of the positive cases were clinically classified as ILI, 31.5% as SARI, and 9.1% as fatal. Influenza A(H1N1)pdm09 cases presented a median age of 35 years old (ranging from 1 to 88 years old), and 52.3% were male and 47.7 were female. Regarding symptomatology, 80.3% showed dyspnea, 36.2% showed respiratory failure, and 28.8% showed oxygen saturation < 95%. In addition, 44.7% also had a previous comorbidity such as obesity, diabetes, pneumopathy, or immunodepression (Table 1). The case inclusion criteria were the availability of sufficient information on the epidemiological forms that would enable their classification into the mentioned clinical groups. This study was approved by the Ethics Committee of Fiocruz, IOC n° 68118417.6.0000.453.470.

### 2.2. Diagnosis of Influenza A(H1N1)pdm09

The majority of the samples had a previous positive result for the detection of influenza A(H1N1)pdm09, which was performed by the state laboratories of the Brazilian influenza surveillance system. A small subset of samples was diagnosed at our laboratory. For that, total RNA was extracted from nasal and oropharyngeal swab samples with a commercial QIAmp viral RNA kit (Qiagen, Germany), following the manufacturer’s guidelines. Afterwards, the RNA was submitted to a one-step real-time RT-PCR reaction with influenza A(H1N1)pdm09-specific oligonucleotides and probes, according to supplier’s conditions (Centers for Disease Control, USA) using Step-One or 7500 Real-Time PCR devices (Applied Biosystems, USA).

### 2.3. OPN Detection in the Respiratory Samples

After extracting the total RNA from the samples, reverse transcription was performed starting from 1 µg of RNA, followed by a treatment with DNAse I (Thermo Fisher Scientific, USA) and RNaseOUT (Thermo Fisher Scientific, USA). The synthesis of the complementary DNA was performed with a Superscript III enzyme (Thermo Fisher Scientific, USA). Oligo dT (Thermo Fisher Scientific, USA) was used as the primer for the cDNA synthesis. Subsequently, to verify the presence of OPN transcripts in the nasopharyngeal samples, we performed conventional PCR with Taq DNA polymerase (Thermo Fisher Scientific, USA) and specific oligonucleotides as follows: OPNa: F 5′ ATCTCCTAGCCCCACAGAAT 3′ and R 5′ CATCAGACTGGTGAGAATCATC 3′; OPNb: F 5′ ATCTCCTAGCCCCAGAGAC 3′ and R 5′ AAAATCAGTGACCAGTTCATCATCAG 3′; OPNc: F 5′ CTGAGGAAAAGCAGAATGCTG 3′ and R 5′ GTCAATGGAGTCCTGGCTGT 3′; OPN4: F 5′ AAGCAGACCCTTCCAAGTAA 3′ and R 5′ ATCAGAGTCGTTCGAGTCAATG 3′; OPN5: F 5′ GATGTACCTACCCCTCCACAACAGATGA 3′ and R 5′ AGTGACCCCCAAGGCAGCTCTATTT 3′; tOPN: F 5′ CCAACGAAAGCCATGACCAC 3′ and R 5′ CTGTGGGGACAACTGGAGTG 3′ (Figure 1). GAPDH transcripts were also evaluated with the following oligonucleotides: F 5′ TGACCCCTTCATTGACCTCA 3′ and R 5′ AGTCCTTCCACGATACCAAA 3′. Importantly, tOPN oligonucleotides encompass all splicing isoforms of OPN transcripts as they bind to exon 6, present in all OPN isoforms. Their amplification protocol started with incubation at 95 °C for 5 min, followed by 35 cycles of 94 °C for 30 s, 50 °C for 30 s, and 72 °C for 30 s, and a final extension step at 72 °C for 5 min [30]. To visualize the amplified amplicons, electrophoresis was performed on 1.5% agarose gels (Invitrogen) with 5 µL of SyBR safe (Thermo Fisher Scientific, USA) in Tris/Borate/EDTA (Sigma, USA). The amplicons were visualized using an ultraviolet transilluminator. 

### 2.4. OPN Transcript Levels

*OPN* transcript expression levels were analyzed by quantitative real-time PCR (qRT-PCR) by using the SyBR Green methodology (Thermo Fisher Scientifics, USA) in Step-One or 7500 equipment (Applied Biosystems, USA), and further analyzed using the ΔΔCT method. For this, 40 ng of cDNA was used in addition to 5 µL of SyBR Green and 2.5 µL of a mix containing the specific oligonucleotides (final concentration of 0.2 mM), as mentioned above. The amplification protocol started with incubation at 50 °C for 2 min and 94 °C for 5 min, followed by 40 cycles of 94 °C for 30 s, 50 °C for 30 s, and 72 °C for 30 s, and a final extension step at 72 °C for 5 min. After that, a melting curve was performed. GAPDH was used as a constitutive normalizing gene for the analysis.

### 2.5. Statistical Analysis

The chi-squared test was used to verify the association between the OPN expression and the clinical groups and severity of symptoms using SPSS software for Windows, version 19.0 (IBM Inc., USA). OPN expression levels were compared between the clinical groups of IAV-infected cases by a one-way ANOVA with Tukey’s post-test for multiple comparisons. Finally, the correlation between the OPN expression and influenza viral load was assessed by a linear regression to calculate the R^2^ and *p*-values. The results were considered to be significant when *p* < 0.05. For those, we used Graph Pad Prism software, version 8.

## 3. Results

### 3.1. tOPN and OPN4 Transcripts Are more Prevalent in Respiratory Samples from Influenza A(H1N1)pdm09 Cases

We investigated the presence of *tOPN* and splicing isoform OPN transcripts in respiratory samples and found that *tOPN* was more prevalently detected in IAV-positive samples when compared with negative control samples (34.1% versus 18.5%, respectively; *p* < 0.05) as well as in fatal IAV in comparison with the non-fatal samples (59.1% versus 30.5%, respectively; *p* < 0.01). Moreover, *OPN4* splicing isoform mRNA was also more prevalently observed in IAV cases than in the negative control group (78.5% versus 66.1%, respectively; *p* = 0.05) and in severe IAV cases in relation to the non-severe ones (85.7% versus 68.2%, respectively; *p* < 0.01) (Table 2).

Of note, the detection of *OPNa* and *OPNc* was observed merely in 8 (4.5%) and 20 (11.4%) IAV samples, respectively, which impaired a robust comparison of their prevalence between the clinically distinct group extracts. Moreover, *OPNb* and *OPN5* were not detected in the analyzed samples.

As we identified an association between *tOPN* and *OPN4* detection and influenza A(H1N1)pdm09 infection, we further investigated the relationship between their prevalence in the cases that also presented severe influenza symptoms such as dyspnea, respiratory failure, and oxygen saturation. Notably, *tOPN* was more frequently detected in the samples from individuals that presented dyspnea (*p* < 0.05). In addition, we found that *OPN4* transcript detection was more predominantly observed in specimens from patients who presented all the aforementioned severity symptoms (*p* < 0.05) (Table 3). These data reinforced that *tOPN* and *OPN4* detection in the respiratory specimens from influenza A(H1N1)pdm09 cases was associated with the IAV infection severity. 

To exclude the involvement of pre-existing comorbidities that would affect the expression of *tOPN* and *OPN4*, we evaluated their detection regardless of influenza subgroups and found that there was no correlation between the presence of comorbidities, smoking, and cancer and the detection of *tOPN* and *OPN4.*

### 3.2. OPN4 Expression Level Is Increased in Respiratory Samples from Fatal Influenza A(H1N1)pdm09 Patients

**Figure 2:**
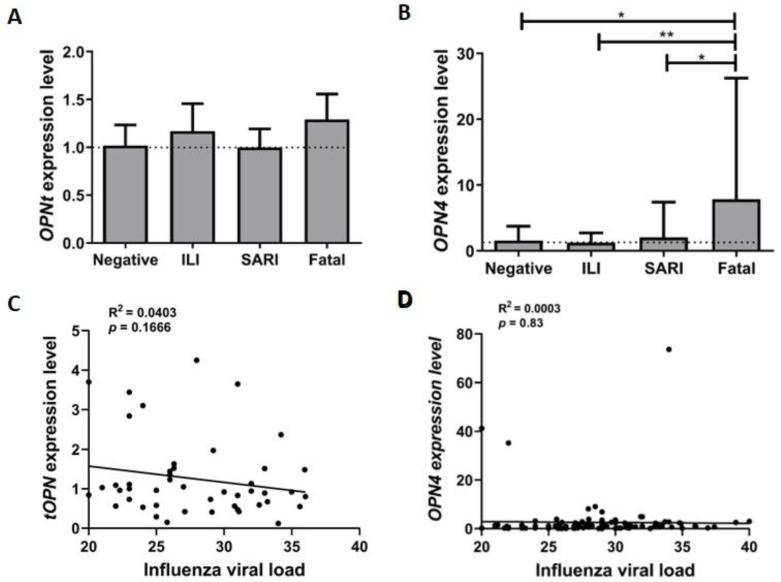
*tOPN* and *OPN4* expression levels and influenza viral load in the different clinical groups. *tOPN* (**A**) *and OPN4* (**B**) expression levels in respiratory specimens from clinically differentially classified influenza A(H1N1)pdm09 cases. Comparison was performed between clinal groups and negative influenza infection (control group), and all groups among themselves. The bars show this comparison. The dotted line demonstrates the starting point from the control group. Statistical analyses were performed using the one-way ANOVA test with Tukey’s post-test. Correlation analysis of *tOPN* (**C**) and *OPN4* (**D**) expression levels and influenza viral load was performed by linear regression. R^2^ and *p*-value were calculated. The analysis of the level of expression of the transcripts was performed by real-time quantitative PCR and ΔΔCT methodology. GAPDH was used as a constitutive normalizing gene for these analyses. ILI: influenza-like illness; SARI: severe acute respiratory infection. * *p* < 0.05; ** *p* < 0.01.

We further determined the *tOPN* and *OPN4* expression levels in the investigated respiratory specimens. We found that *tOPN* expression levels were similar in the distinct IAV clinical subgroups and negative controls analyzed (Figure 2A). As opposed to that, *OPN4* showed higher expression levels in the IAV-fatal group when compared with the negative, ILI, and SARI cases (*p* < 0.05, *p* < 0.01, and *p* < 0.05, respectively) (Figure 2B). Moreover, we also assessed the correlation between the *tOPN* and *OPN4* expression levels and influenza viral load in the clinical samples, regardless of their severity, which revealed no significant association (R^2^ = 0.0402, *p* = 0.16 and R^2^ = 0.0004, *p* = 0.83, respectively) (Figure 2C,D).

## 4. Discussion

IAV infections cause a significant disease burden annually and have been the source of pandemics over the centuries. Clinically, the disease can range from acute mild to a serious condition that can progress to fatality. The unfavorable outcome is more frequently observed in the individuals included in the classical risk groups when compared with healthy individuals. Noteworthy, healthy individuals bearing no pre-existing conditions that progress poorly correspond with a subgroup that would highly benefit from the detection of biomarkers of disease progression. Therefore, it is necessary to identify host factors that could be used for this purpose as an auxiliary tool in the follow-up, anticipation of the disease evolution, and guiding of the specific clinical management in these individuals. In this scenario, this work aimed to investigate the modulation of *tOPN* and OPN isoform transcript expressions by IAV infection. They were selected as interesting targets and promising candidates as they are involved in multiple steps of immune responses and have been previously reported as altered after other viral infections [20,31,32]. 

Herein, we described that *tOPN* and *OPN4* were more frequently detected in respiratory samples from influenza A(H1N1)pdm09 cases. Moreover, we related the upregulation of *OPN4* expression in respiratory samples from fatal influenza A(H1N1)pdm09 patients. Corroborating these data, a recent report also showed that the OPN transcript expression level is increased in human blood samples from IAV cases compared with healthy individuals [22]. This result was confirmed with in vivo and in vitro models after IAV infections [22]. In addition, a previous report showed that OPN serum levels were higher in influenza A(H7N9) samples compared with healthy volunteers, despite OPN serum levels being similar in A(H1N1) patients with mild symptoms and healthy volunteers [33]. The relationship of OPN modulation and the severity of influenza infections may be associated with their implication in the activation of the immune response. It was recently demonstrated that microglial cells infected with influenza A(H1N1) demonstrated an upregulation of *OPN* expression [34] and the induction of cytokine and chemokine production and apoptosis [34]. OPN also aggravated lung inflammation by the induction of macrophage necroptosis and a reduction in IAV clearance [22]. Macrophages have a relevant role during influenza infections [35]. Initially, they can be polarized both by the signaling cascades activated by the contact with pathogens or with cytokines secreted by alveolar epithelial cells (AECs) [36]. As macrophages are large producers of OPN, it is suggested that their activation following influenza-infected AECs increases OPN levels, culminating in a positive feedback with the recruitment of more macrophages to the lung, which is often one of the hallmarks of a severe influenza infection [37]. 

Moreover, our data showed no relationship between *tOPN* and *OPN4* levels and the influenza A(H1N1)pdm09 viral load. This was contradictory to a former report, which showed that the OPN level increased during RSV infection and was proportional to the viral load in an in vivo model [21]. However, the mechanism by which OPN participated in the increase in viral load observed in this study was not clear. OPN acts in the innate immune response modulation, being produced after the initial phase of the infection [38]. The samples used in this study were collected within a period of up to 7 days after the onset of symptoms. This variation in the collection period may be correlated with the lack of association between the OPN expression level and viral load. Further studies may evaluate the time-course of the OPN expression and viral load over the course of an influenza infection to elucidate the gaps regarding this interaction. 

In our study, we included a control group comprising patients who were characterized as negative for an influenza infection despite presenting ILI-like symptoms suggestive of a respiratory infection with a distinct pathogen. The inclusion of this control group increased the specificity of our findings in relation to the abnormal presence of OPN isoforms in the respiratory influenza A(H1N1)pdm09 samples. However, in our analyses, there was no correlation between the presence of comorbidities and *tOPN* and *OPN4,* which also demonstrates that the modulation of OPN levels could be more precisely associated with an influenza infection and unrelated to the patient’s pre-existing conditions. However, as OPN can be secreted, it may be less advantageous to assess OPN transcript levels in nasal and oropharyngeal swab samples than evaluating its protein level in other sample types such as bronchoalveolar lavage fluid, plasma, and urine, which would be more feasible for its clinical application. Further OPN analyses in additional cohorts and distinct samples are being conducted to address this question. The identification of specific expression patterns of human biomarkers during infections and disease progression could add great value to the clinical management of IAV infections [39,40]. 

In conclusion, our results demonstrated that *tOPN* and *OPN4* expression was more prevalent during influenza A(H1N1)pdm09 infections, which was also associated with unfavorable clinical outcomes and the severity of respiratory symptoms. Further studies will better investigate the mechanisms involved in this modulation and the potential application as an influenza infection prognostic marker.

## Figures and Tables

**Table 1:** Clinical and epidemiological characteristics of the patients.

Characteristics	Negative	ILI	SARI	Fatal	Total
Number of samples	65	78	76	22	241
Age (years), median ± SD	17 ± 30.9	28 ± 22.6	46 ± 23.7	49 ± 14.9	35 ± 25.7
Male, *n* (%)	34 (53.1)	47 (60.3)	35 (46.0)	10 (45.4)	126 (52.3)
Female, *n* (%)	31 (46.9)	31 (39.7)	41 (54.0)	12 (54.6)	115 (47.7)
**Symptoms, *n/N* (%)**					
Dyspnea	27/50 (54.0)	NA	71/76 (93.4)	16/16 (100)	114/142 (80.3)
Respiratory distress	23/65 (35.4)	NA	36/76 (47.4)	4/22 (18.2)	59/163 (36.2)
Oxygen saturation < 95%	15/65 (23.1)	NA	28/76 (36.8)	4/22 (18.2)	47/163 (28.8)
Comorbidities, *n/N* (%) *	11/28 (39.3)	8/23 (34.8)	24/48 (50.0)	3/4 (75.0)	46/103 (44.7)

All comparisons were performed between the clinical groups. SD: standard deviation; ILI: influenza-like illness; SARI: severe acute respiratory infection; NA: not applicable; n: number of cases with the referred characteristic; N: number of cases for which the information is available. * Obesity, diabetes, pneumopathy, immunodepression, arterial hypertension, and cardiovascular diseases.

**Table 2:** Frequency of detection of *tOPN* and OPN isoforms in respiratory samples, according to influenza A(H1N1) positiveness and disease severity clinical groups.

mRNA Detection	Groups	*n/N* (%)	*p*-Value
*tOPN*	Negative	12/65 (18.5)	<0.05
	A(H1N1)pdm09	60/176 (34.1)	
	ILI	22/78 (28.2)	0.14
	Severe (SARI + fatal)	38/98 (38.8)	
	Non-fatal (ILI + SARI)	47/154 (30.5)	<0.01
	Fatal	13/22 (59.1)	
*OPNa*	Negative	2/65 (3.1)	0.61
	A(H1N1)pdm09	8/176 (4.5)	
	ILI	2/78 (2.6)	0.26
	Severe (SARI + fatal)	6/98 (6.1)	
	Non-fatal (ILI + SARI)	5/154 (3.2)	0.03
	Fatal	5/22 (13.6)	
*OPNb*	Negative	0/65 (0)	NA
	A(H1N1)pdm09	0/176 (0)	
	ILI	0/78 (0)	NA
	Severe (SARI + fatal)	0/98 (0)	
	Non-fatal (ILI + SARI)	0/154 (0)	NA
	Fatal	0/22 (0)	
*OPNc*	Negative	8/65 (12.3)	0.84
	A(H1N1)pdm09	20/176 (11.4)	
	ILI	10/78 (12.8)	0.59
	Severe (SARI + fatal)	10/98 (10.2)	
	Non-fatal (ILI + SARI)	16/154 (10.4)	0.28
	Fatal	4/22 (18.2)	
*OPN4*	Negative	43/65 (66.1)	0.05
	A(H1N1)pdm09	138/176 (78.4)	
	ILI	54/78 (69.2)	<0.01
	Severe (SARI + fatal)	84/98 (85.7)	
	Non-fatal (ILI + SARI)	119/154 (77.3)	0.33
	Fatal	19/22 (86.4)	
*OPN5*	Negative	0/65 (0)	NA
	A(H1N1)pdm09	0/176 (0)	
	ILI	0/78 (0)	NA
	Severe (SARI + fatal)	0/98 (0)	
	Non-fatal (ILI + SARI)	0/154 (0)	NA
	Fatal	0/22 (86.4)	

Statistical analysis was performed with the chi-squared test. Comparison of the following groups was performed: negative cases versus positive influenza A(H1N1)pdm09 cases; ILI versus severe (SARI + fatal); and non-fatal (ILI+SARI) versus fatal cases. *tOPN*: total OPN; ILI: influenza-like illness; SARI: severe acute respiratory infection; n: number of positive samples; N: number of cases for which the information is available; NA: not applicable.

**Table 3:** Frequency of detection of *tOPN* and *OPN4* isoforms in severe influenza A(H1N1)pdm09 cases presenting dyspnea, respiratory failure, and oxygen saturation < 95%.

mRNA Detection	Dyspnea			Respiratory Failure			Oxygen Saturation < 95%		
	Positive	Negative		Positive	Negative		Positive	Negative	
	*n/N* (%)	*n/N* (%)	*p*-Value	*n/N* (%)	*n/N* (%)	*p*-Value	*n/N* (%)	*n/N* (%)	*p*-Value
*tOPN*	32/87 (36.8)	6/32 (18.7)	0.04	4/40 (35.0)	46/136 (33.8)	0.79	9/32 (28.1)	51/144 (35.4)	0.41
*OPN4*	75/87 (86.2)	21/32 (65.6)	0.01	36/40 (90.0)	102/136 (75.0)	0.02	30/32 (93.7)	108/144 (75.0)	0.01

Statistical analysis was performed with the chi-squared test. Comparison of the following groups was performed: positive for the presence of the specified mRNA (tOPN or OPN4) in the groups positive or negative for the severity symptom. *tOPN*: total OPN; N: number of samples with symptoms of severity; ILI: influenza-like illness; SARI: severe acute respiratory infection.

## Data Availability

The data presented in this study are available on request from the corresponding author.
